# Fucoxanthin provides neuroprotection in models of traumatic brain injury via the Nrf2-ARE and Nrf2-autophagy pathways

**DOI:** 10.1038/srep46763

**Published:** 2017-04-21

**Authors:** Li Zhang, Handong Wang, Youwu Fan, Yongyue Gao, Xiang Li, Zhigang Hu, Ke Ding, Yujie Wang, Xiaoliang Wang

**Affiliations:** 1 Department of Neurosurgery, Jinling Hospital, School of Medicine, Nanjing University, Nanjing, Jiangsu Province, China

## Abstract

Fucoxanthin is abundant in seaweed and is considered as a powerful antioxidant. It has been proposed to possess anti-cancer, anti-obesity and anti-diabetes effects. However, its roles in brain injury models have not been fully understood. The objective of this study was to investigate the neuroprotection of fucoxanthin in models of traumatic brain injury (TBI) and the role of the nuclear factor erythroid 2-related factor 2 (Nrf2)-antioxidant-response element (ARE) and Nrf2-autophagy pathways in the putative neuroprotection. We found that fucoxanthin alleviated TBI-induced secondary brain injury, including neurological deficits, cerebral edema, brain lesion and neuronal apoptosis. Moreover, the up-regulation of malondialdehyde (MDA) and the activity of glutathione peroxidase (GPx) were reversed by fucoxanthin treatment. Furthermore, our *in vitro* studies demonstrated that fucoxanthin increased the neuron survival and reduced the reactive oxygen species (ROS) level. In addition, fucoxanthin activated the Nrf2-ARE pathway and autophagy both *in vivo* and *in vitro*, which was proven by the results of immunohistochemistry, western blot and electrophoretic mobility shift assay (EMSA). However, fucoxanthin failed to provide neuroprotection and activated autophagy following TBI in Nrf2^−/−^ mice. In conclusion, our studies indicated that fucoxanthin provided neuroprotective effects in models of TBI, potentially via regulation of the Nrf2-ARE and Nrf2-autophagy pathways.

Traumatic brain injury (TBI) is a leading cause of death and disability in the young aged population and it has been a major public health problem in modern society^1^. TBI causes primary mechanical injury of brain cells and initiates secondary damage such as oxidative stress, inflammation and apoptosis that occurs immediately after the primary damage. Although the primary brain injury is the major factor determining the outcomes, the secondary brain injury exacerbates the damage of TBI^2^,^3^. Patients who suffer with TBI always end up with poor prognosis despite decades of concerted efforts and advances in surgery and therapeutic drugs. Therefore, in order to reduce the high disease burden, the development of new and effective therapeutic strategies is urgently needed.

Nuclear factor erythroid 2-related factor 2 (Nrf2) is a basic leucine zipper redox-sensitive transcription factor that controls the redox state of cell in harmful stresses^4^. Under basal conditions, Nrf2 is localized in the cytoplasm and anchored by its inhibitor, Kelch-like ECH-associated protein 1 (Keap1)^5^. However, under conditions of oxidative or xenobiotic stress, Nrf2 dissociates from Keap1, translocates into the nucleus and regulates the expression of antioxidant genes such as heme oxygenase-1 (HO-1) and NADPH:quinine oxidoreductase-1 (NQO-1) via interaction with the antioxidant response element (ARE)^6^. In addition, recent reports have demonstrated that Nrf2 could regulate autophagy formation, further promoting its protective effects^7^,^8^. To date, Nrf2 has been proven to be a protective molecular in many pathological processes and activated in various central nervous system (CNS) diseases^9^,^10^, including TBI^11^.

Fucoxanthin is the most abundant marine carotenoid extraction from seaweeds, contributing more than 10% of the total production of carotenoids in nature^12^. Because of its special functional groups, including an unusual allenic bond and a 5,6-monoepoxide structure, fucoxanthin exhibits a variety of pharmacological properties such as reducing oxidative stress and repressing inflammation reaction^13^. Previous studies have focused on the role of fucoxanthin in cancer treatment and health protection. For example, fucoxanthin was shown to augment apoptosis, reduce cell proliferation, migration and invasion in human glioblastoma cell lines^14^. Furthermore, fucoxanthin in diet significantly reduced weight gain in animals through increasing fatty acid oxidation and heart production in white adipose tissue^15^. Unfortunately, none reports so far have claimed its role in brain injury models, particularly in the models of TBI. The present study was aimed to investigate the neuroprotective effects of fucoxanthin after TBI and the possible role of Nrf2-ARE and Nrf2-autophagy pathways in the putative neuroprotection of fucoxanthin.

## Results

### General observations and the mortality rate of mice

All the *in vitro* and *in vivo* measurements in our experiments were conducted by a blinded investigator. Total 455 wild type mice and 116 Nrf2-deficient mice were used in this study, among them 42 mice (32 wild type mice and 10 Nrf2-deficient mice) died during the operation. The mice that died during the operation were not included in the mortality calculations and excluded from further analysis. The mortality of mice within 24 h in each group was as follows: sham group 0% (0 of 55 mice), TBI group 15.4% (10 of 65 mice), TBI + vehicle group 12.7% (8 of 63 mice), TBI + 50 mg/kg fucoxanthin intragastric (i.g.) group 14.3% (4 of 28 mice), TBI + 100 mg/kg fucoxanthin i.g. group 15.4% (10 of 65 mice), TBI + 200 mg/kg fucoxanthin i.g. group 14.3% (4 of 28 mice), TBI + 0.01 mmol/L fucoxanthin intracerebroventricular (i.c.v.) group 11.1% (3 of 27 mice), TBI + 0.05 mmol/L fucoxanthin i.c.v. group 14.1% (9 of 64 mice), TBI + 0.1 mmol/L fucoxanthin i.c.v. group 14.3% (4 of 28 mice), TBI + vehicle group (Nrf2^−/−^ mice) 14.3% (5 of 35 mice), TBI + 100 mg/kg fucoxanthin i.g. group (Nrf2^−/−^ mice) 14.3% (5 of 35 mice), TBI + 0.05 mmol/L fucoxanthin i.c.v. group (Nrf2^−/−^ mice) 16.7% (6 of 36 mice). There were no significant differences in mortality between groups of wild type mice and Nrf2-deficient mice (data not shown).

### Fucoxanthin provided neuroprotection after TBI

**Figure 1:**
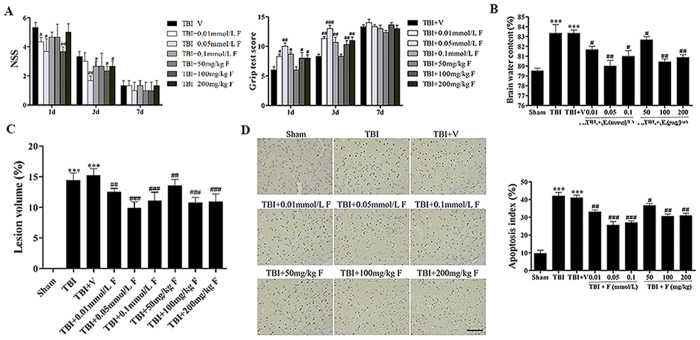
Administration of fucoxanthin protected mice against secondary brain injury after TBI. (**A**,**B**) Mice were subjected to TBI and then received 50 mg/kg, 100 mg/kg, 200 mg/kg of fucoxanthin i.g. administration or 0.01 mmol/L, 0.05 mmol/L, 0.1 mmol/L of fucoxanthin i.c.v. injection or vehicle 30 min after TBI. NSS and Grip test score was evaluated at 1, 3, and 7 days after TBI while brain water content was examined at 1 day after TBI. (**A**) All doses of fucoxanthin had an improved motor performance within 3 days; however, larger doses such as 200 mg/kg and 0.1 mmol/L did not exhibit a better neuroprotection. This effect was no longer significant at 7 days after TBI. n = 6 per group. (**B**) Mice subjected to TBI or treated with vehicle had an increased brain water content as compared with the sham group. Brain water content was significantly lower in the groups with treatment of fucoxanthin than the vehicle-treated group. Moreover, doses of 100 mg/kg and 0.05 mmol/L had the best effect in relieving brain edema. n = 6 each group. (**C**) TBI-induced profound tissue loss of the brain was reversed by fucoxanthin, and doses of 100 mg/kg and 0.05 mmol/L had the best effect. (**D**) Apoptotic index was determined using TUNEL assays 1 day after TBI. The apoptotic index was significantly higher after TBI compared to the sham group. Fucoxanthin treatment significantly decreased the percentage of apoptotic cells after TBI, and doses of 100 mg/kg and 0.05 mmol/L had the best effect in reducing apoptosis induced by TBI. n = 6 each group. Data are presented as mean ± SEM; ***p < 0.001 versus sham group; ^#^*p* < 0.05, ^##^*p* < 0.01, ^###^*p* < 0.001 versus TBI + vehicle group. Scale bar: 50 mm.

To determine whether fucoxanthin provided neuroprotection after TBI. We set nine groups as follows: sham, TBI, TBI + vehicle, TBI + fucoxanthin intragastric (i.g.) administration (three different dose groups: 50 mg/kg, 100 mg/kg, 200 mg/kg), TBI + fucoxanthin intracerebroventricular (i.c.v.) injection (three different dose groups: 0.01 mmol/L, 0.05 mmol/L, 0.1 mmol/L). We firstly used the neurological severity score (NSS) and grip test to evaluate the motor performance of mice following TBI. All mice were trained 1 day before TBI. The sham group showed no difference among various time points and there is no difference between the TBI group and the vehicle-treated group (data no shown). As showed in Fig. 1A, the motor performance of fucoxanthin-treated mice was significantly better than that of the vehicle-treated mice at 1 d. In addition, at 3 d, a significant difference was still detectable. However, there was no significant difference between the TBI + vehicle group and TBI + fucoxanthin group at 7 d (*p* > 0.05).

We also used brain water content to confirm the neuroprotection of fucoxanthin. As an indicator of brain edema, brain water content was usually measured at 1 d after TBI according to the previous study^16^. Compared with the sham group, a significant increase in brain water content was observed at 1 d post-TBI (Fig. 1B). However, the brain water content was obviously reduced by fucoxanthin treatment.

Then we determined whether fucoxanthin could affect the cortical lesion volume after TBI. As shown in Fig. 1C, TBI caused profound tissue loss of the brain. However, fucoxanthin decreased lesion volume induced by TBI. Collectively, our results suggested that fucoxanthin was neuroprotective against TBI. Importantly, our experiments above indicated that larger doses such as 200 mg/kg and 0.1 mmol/L did not exhibit a better neuroprotection (Fig. 1A,B and C).

### Fucoxanthin reduced TBI-induced apoptosis

To examine whether the neuroprotective effect of fucoxanthin in TBI was bound with apoptosis, we firstly used Terminal deoxynucleotidyl transferase-mediated dUTP nick 3′-end labeling (TUNEL) staining to detect the neural cell apoptosis. Results revealed that few TUNEL-positive cells were found in the sham group, while a large number of TUNEL-positive cells were found in TBI group (there is no difference between the TBI group and the vehicle-treated group, *p* > 0.05). However, fucoxanthin treatment significantly decreased the number of TUNEL-positive cells (Fig. 1D). Consistent with the NSS, grip test, lesion volume measurements and brain water content, doses of 100 mg/kg and 0.05 mmol/L had the best effect in reducing apoptosis induced by TBI. Taken together, our data demonstrated that fucoxanthin had the potential to ameliorate the secondary brain insult following TBI, and suggested that doses of 100 mg/kg and 0.05 mmol/L exhibited the best effect, which we used in the following experiments.

**Figure 2:**
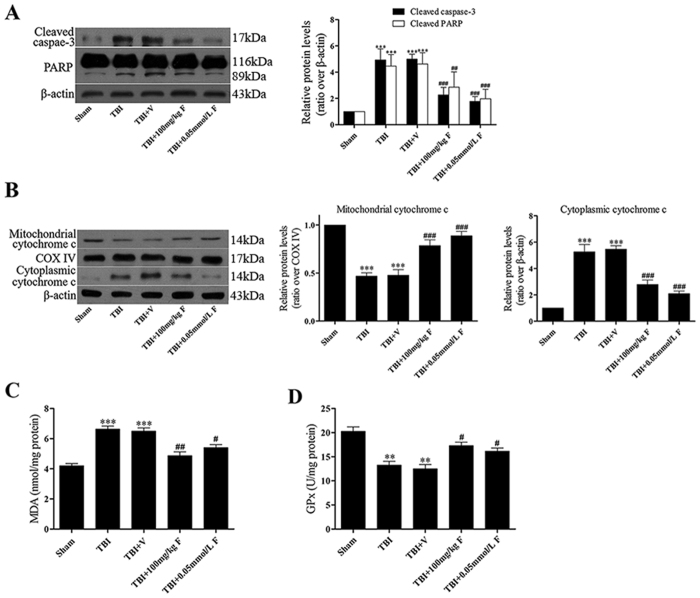
Fucoxanthin suppressed TBI-induced apoptosis and oxidative stress. Mice were subjected to TBI and treated with 100 mg/kg and 0.05 mmol/L fucoxanthin or vehicle 30 min after TBI. Ipsilateral brain tissues were collected 1 day after TBI. (**A**,**B**) The protein levels of cleaved caspase-3, poly (ADP-ribose) polymerase (PARP) and cytochrome c were measured by western blot. Fucoxanthin significantly decreased apoptosis induced by TBI. Oxidative stress is represented by the level of MDA and the activity of GPx. (**C**) MDA was significantly increased after TBI compared to the sham group, while treatment with fucoxanthin significantly restored this change. (**D**) Inversely, TBI significantly lowered the activity of GPx; however, the activity of GPx was raised after treatment with fucoxanthin. Data are presented as mean ± SEM, n = 6 per group; **p < 0.01, ***p < 0.001 versus sham group; ^#^*p* < 0.05, ^##^*p* < 0.01, ^###^*p* < 0.001 versus TBI + vehicle group. COX IV was used as a loading control for mitochondria extracts. β-actin was used as a loading control for cytoplasmic and whole-cell extracts.

To further elucidate the effects of fucoxanthin on TBI-induced apoptosis, we examined several apoptosis indicators such as caspase-3, poly (ADP-ribose) polymerase (PARP) and cytochrome c. The observation showed that the cleaved caspase-3, cleaved PARP and cytoplasmic cytochrome c were increased while the mitochondrial cytochrome c was decreased following TBI (Fig. 2A and B). However, compare with the vehicle-treated group, fucoxanthin treatment significantly suppressed the TBI-triggered apoptosis (Fig. 2A and B), indicating that fucoxanthin was able to reduce cell death in the cortical contusion after TBI.

### Fucoxanthin reduced oxidative stress in injured brains

Then, we determined the oxidative stress levels in brain tissue. Malondialdehyde (MDA) reflects the level of lipid peroxidation and glutathione peroxidase (GPx) catalyzes the reactions of reduced glutathione. Our results showed that MDA was increased in the TBI and the TBI + vehicle groups (Fig. 2C). Treatment of fucoxanthin significantly decreased the generation of MDA (Fig. 2C). Conversely, GPx decreased after TBI, while fucoxanthin obviously activated GPx (Fig. 2C), confirming the protective role of fucoxanthin to ameliorate the secondary brain insult following TBI.

### Fucoxanthin induced autophagy after TBI

**Figure 3:**
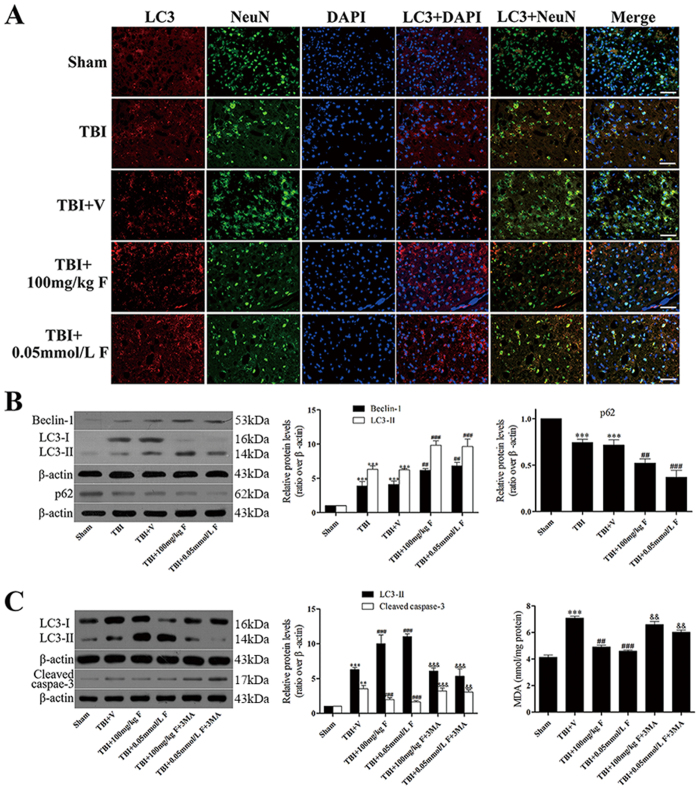
Fucoxanthin activated autophagy after TBI. (**A**) Representative images of immunofluorescence for LC3 surrounding the injured cortex. LC3 punctate dots were observed in the cytoplasm by immunofluorescent staining of LC3 (red). Neuron cells and nuclei are labeled with NeuN (green) and DAPI (blue), respectively. Magnification: 40 x. Scale bar: 50 mm. (**B**) Mice brain tissues were collected 1 day after TBI in different groups, and the expression of LC3, Beclin-1 and p62 was measured by western blot. Fucoxanthin treatment significantly increased the level of LC3-II and Beclin-1 while decreasing the level of p62 after TBI. (**C**) 3-MA (400 nM) was injected i.c.v. 30 min before TBI. Mice were then subjected to TBI and treatment of fucoxanthin 30 min after TBI. Pretreatment with 3-MA significantly attenuated fucoxanthin-induced activation of autophagy and suppression of apoptosis and oxidative stress in the ipsilateral cortex. Data are presented as mean ± SEM, n = 6 per group; **p < 0.01, ***p < 0.001 versus sham group; ^#^*p* < 0.05, ^##^*p* < 0.01 versus TBI + vehicle group; ^&&^*p* < 0.01, ^&&&^*p* < 0.001 versus TBI + fucoxanthin group. β-actin was used as a loading control.

Recently, reports have shown that many drugs can provide neuroprotection in brain injury models through activation of autophagy. Therefore, we determined whether the autophagy pathway was involved in fucoxanthin’s neuroprotection. We firstly used immunofluorescence, results of the fluorescence microscopy revealed that the TBI groups exhibited a significant increase in the number of neurons with microtubule-associated protein 1 light chain 3 (LC3) punctate dots compared to the sham groups (Fig. 3A). In addition, the LC3 punctate dots were further increased after fucoxanthin treatment (Fig. 3A), indicating an induction of autophagy. To further confirm that autophagy was induced, we analyzed the expression of three key autophagic protein Beclin-1 (Atg6), LC3 (Atg8) and p62. During the process of autophagy, Beclin-1 is necessary for the recruitment of other Atg proteins and LC3 is essential for the autophagy formation. p62 is a selective substrate of autophagy and serves as a common readout of autophagic activity. We found that TBI increased the expression of Beclin-1 and LC3-II while decreased the expression of p62 (Fig. 3B), suggesting that autophagy was activated after TBI. Furthermore, the TBI-induced autophagy was enhanced by fucoxanthin, as indicated by further increased expression of Beclin-1, LC3-II and decreased expression of p62 (Fig. 3B). These data demonstrated that fucoxanthin could promote TBI-induced autophagy. Next, to explore the role of autophagy in the protective effects of fucoxanthin following TBI, we used the autophagy inhibitor 3-Methyladenine (3-MA). We found that combined treatment with fucoxanthin and 3-MA significantly reversed fucoxanthin-triggered induction of autophagy, inhibition of apoptosis and suppression of oxidative stress (Fig. 3C), showing that fucoxanthin suppressed TBI-induced apoptosis and oxidative stress via activation of autophagy.

### Fucoxanthin promoted Nrf2 nuclear translocation and enhanced Nrf2-ARE binding

**Figure 4:**
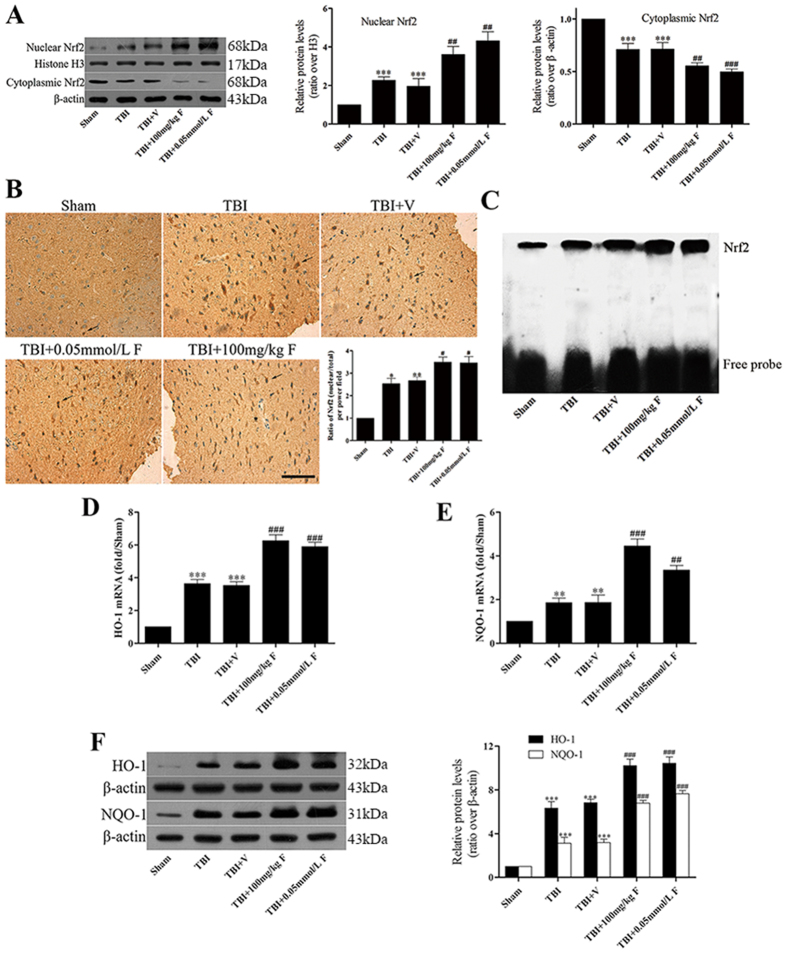
Fucoxanthin activated the Nrf2-ARE pathway. (**A**) Mice brain tissues were collected 1 day after TBI in different groups and the Nrf2 levels in both cytoplasm and nucleus were measured by western blot. Fucoxanthin significantly increased the level of Nrf2 in nucleus and decreased the level of Nrf2 in their counterpart cytoplasm. (**B**) Representative photomicrographs showing Nrf2 immunohistochemistry of tissue from different groups 1 day after TBI. As compared with sham group, the TBI group presented a morphology with Nrf2 concentrated in the nucleus, and after treatment with fucoxanthin, this concentration morphology was more apparent. (**C**) Based on EMSA, Nrf2-ARE-binding activity was enhanced after TBI and substantially increased after treatment with fucoxanthin. (**D**) HO-1 mRNA was elevated after TBI and was further increased with administration of fucoxanthin. (**E**) Similarly, NQO-1 mRNA was elevated after TBI and was further increased with administration of fucoxanthin. (**F**) Both HO-1 and NQO-1proteins were upregulated after TBI and fucoxanthin further increased their expression in brain tissue. Data are presented as mean ± SEM, n = 6 per group; *p < 0.05, **p < 0.01, ***p < 0.001 versus sham group; ^#^*p* < 0.05, ^##^*p* < 0.01, ^###^*p* < 0.001 versus TBI + vehicle group. Scale bar: 50 mm. H3 was used as a loading control for nuclear extracts. β-actin was used as a loading control for cytoplasmic extracts.

The activation of Nrf2 has been reported to regulate autophagy, apoptosis and oxidative stress. So, we examined whether Nrf2 was involved in the neuroprotective role of fucoxanthin. Firstly, we examined the expression of Nrf2 by western blot. Results showed that compared with the sham group, TBI induced the nuclear location of Nrf2 (Fig. 4A). In addition, compared with the TBI and TBI + vehicle-treated groups, the fucoxanthin-treated group significantly increased the nuclear location of Nrf2 and decreased the cytoplasm location of Nrf2 (Fig. 4A). This effect was also confirmed by immunohistochemistry. As seen in Fig. 4B, TBI enhanced Nrf2 concentration in the nucleus while fucoxanthin administration further enhanced the expression of Nrf2 in the nucleus. Consistent with these observations, EMSA results also indicated that treatment of fucoxanthin enhanced nuclear protein Nrf2 binding to ARE (Fig. 4C). These data suggested that fucoxanthin can promote Nrf2 translocation from cytoplasm to nucleus, thereby obtaining elevated binding ability to the downstream genes.

### Fucoxanthin up-regulated the expression of Nrf2 downstream proteins

Nrf2 has been reported to induce the expression of antioxidant enzyme such as HO-1 and NQO-1 through binding to the ARE in the promoter of antioxidant genes, therefore we hypothesized that fucoxanthin might regulate the Nrf2-ARE pathway. Our results have shown that fucoxanthin could activate Nrf2, we then investigated the expression of HO-1 and NQO-1. Figure 4D,E and F showed that the mRNA and protein levels of HO-1 and NQO-1 were up-regulated after TBI. Moreover, fucoxanthin treatment further enhanced the mRNA and protein levels of HO-1 and NQO-1 as compared with TBI and TBI + vehicle groups (Fig. 4D,E and F).

### Protective effects of fucoxanthin in primary cultured neuron

**Figure 5:**
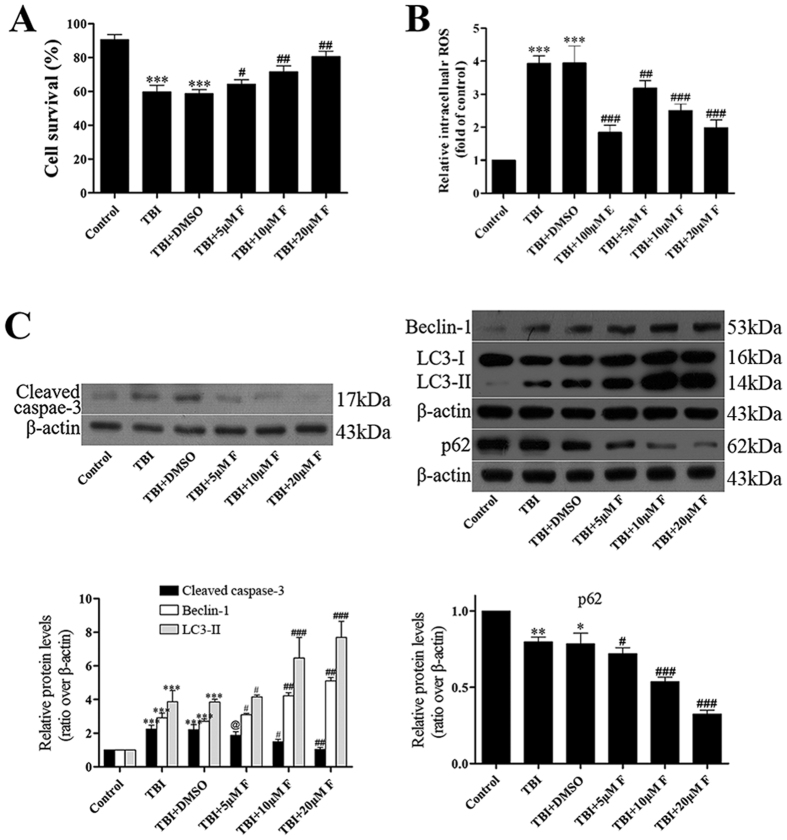
Fucoxanthin protected primary cultured neurons from TBI. (**A**) Primary cortical neurons were subjected to scratch injury and then treated with 5, 10 or 20 μM fucoxanthin or DMSO for 1 day. The LDH release assay was used to evaluate cell viability. The percentage of survival cells significantly decreased after TBI compared to the control group. Fucoxanthin treatment significantly increased survival cells after TBI. (**B**) Fucoxanthin repressed the production of ROS in primary cultured cells after TBI. Cells were subjected to scratch injury and subsequently treated with 100 μM edaravone or 5, 10 or 20 μM fucoxanthin or DMSO for 1 day. Then cells were incubated with DCFH-DA and subjected to fluorescence spectrophotometer analysis. The intracellular ROS was significantly increased after TBI compared to the sham group, and administration of edaravone or fucoxanthin significantly repressed ROS production as compared to the TBI + DMSO group. (**C**) Fucoxanthin inhibited apoptosis and activated autophagy in primary cultured neurons. Primary cortical neurons were subjected to scratch injury and then treated with 5, 10 or 20 μM fucoxanthin or DMSO for 1 day, the expression of cleaved caspase-3, Beclin-1, LC3 and p62 was measured by western blot. Fucoxanthin significantly decreased the expression of cleaved caspase-3 and p62 while increased the expression of Beclin-1 and LC3-II. Data are presented as mean ± SEM, n = 6 per group; *p < 0.05, **p < 0.01, ***p < 0.001 versus control group; ^#^*p* < 0.05, ^##^*p* < 0.01, ^###^*p* < 0.001 versus TBI + DMSO group; ^@^*p* > 0.05 versus TBI + DMSO group. β-actin was used as a loading control.

We further confirmed the neuroprotective effects of fucoxanthin in primary cultured neurons. We firstly performed lactate dehydrogenase (LDH) release assay on cultured neurons which underwent scratch with different concentrations of fucoxanthin. We found that fucoxanthin evidently increased cell survival in a dose-dependent manner (Fig. 5A). Next, to understand the effects of fucoxanthin on intracellular reactive oxygen species (ROS) production, we used 2′, 7′-dichlorodihydrofluorescein diacetate (DCF-DA) fluorescence to measure intracellular ROS levels. The ratios of the ROS levels to the control group were expressed as a percentage of fluorescence intensity. Besides fucoxanthin, we used a verified free radical scavenger, edaravone, as a positive control. As shown in Fig. 5B, the ROS levels in the damaged cells were increased compared with the control group. However, when edaravone or fucoxanthin was added, the ratios of intracellular ROS levels of cells were significantly decrease. Notably, the anti-antioxidation effect of 20 μM fucoxanthin was similar to that of edaravone, demonstrating that fucoxanthin could prevent the accumulation of ROS induced by TBI. In addition, the apoptosis markers such as cleaved caspase-3 and autophagy markers such as Beclin-1, LC3 and p62 were also measured by western blot. Results showed that compared with control cells, cleaved caspase-3, Beclin-1 and LC3-II was up-regulated while p62 was down-regulated in damaged cells. Fucoxanthin treatment further increased the expression of Beclin-1, LC3-II and decreased the expression of cleaved caspase-3, p62 (Fig. 5C). These data indicated that fucoxanthin could increase cell survival, suppress oxidative stress and apoptosis, and activate autophagy in TBI model of primary cultured neuron.

### Fucoxanthin activated the Nrf2-ARE pathway *in vitro*

**Figure 6:**
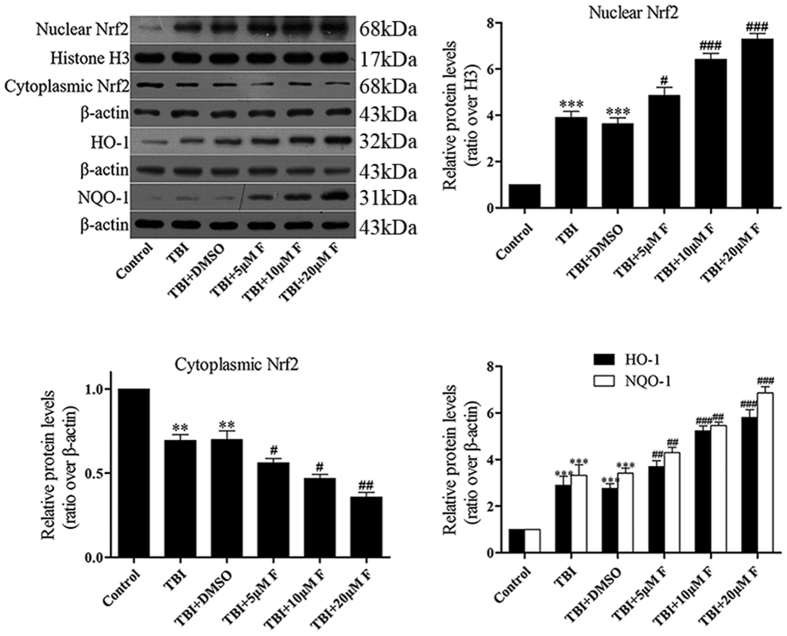
Fucoxanthin promoted Nrf2 translocation from cytoplasm to nucleus and actives the proteins downstream in primary cultured neurons. Fucoxanthin significantly increased the level of Nrf2 in the nucleus and consequently reduced the level of Nrf2 in the cytoplasm. Elevated levels of HO-1 and NQO-1 which were downstream of Nrf2 were examined after TBI, and fucoxanthin treatment significantly enhanced their expression as compared to DMSO treated. Data are presented as mean ± SEM, n = 6 per group; **p < 0.01, ***p < 0.001 versus control group; ^#^*p* < 0.05, ^##^*p* < 0.01, ^###^*p* < 0.001 versus TBI + DMSO group. H3 was used as a loading control for nuclear extracts. β-actin was used as a loading control for cytoplasmic extracts.

Western blot analysis showed that the nuclear Nrf2 increased after TBI and fucoxanthin further enhanced the nuclear Nrf2 in a dose-dependent manner. By contrast, the cytoplasmic Nrf2 was decreased. Furthermore, fucoxanthin treatment increased the expression of downstream proteins HO-1 and NQO-1 in a dose-dependent manner (Fig. 6). Taken together, our *in vitro* results indicated that fucoxanthin provided neuroprotection in *in vitro* TBI model and activated the Nrf2-ARE pathway.

### Fucoxanthin failed to protect brain injury and activate autophagy in Nrf2-deficient mice

**Figure 7:**
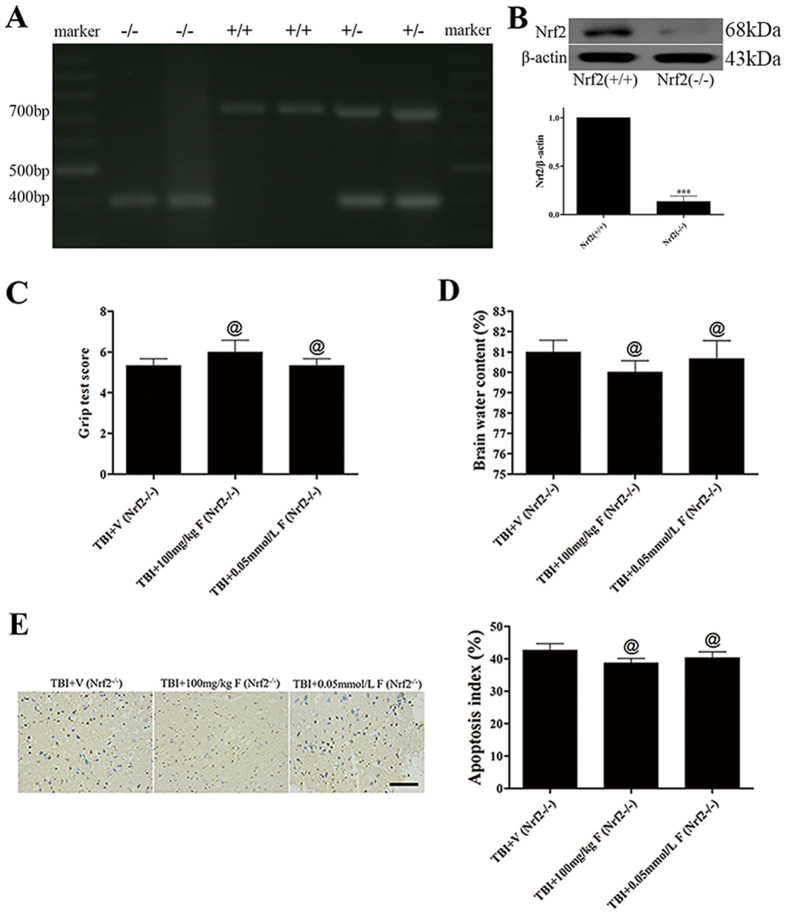
Fucoxanthin failed to provide neuroprotection against TBI in Nrf2^−/−^ mice. (**A**) The results of genotype identification by RT-PCR. (**B**) Nrf2 knockout markedly decreased the level of Nrf2 at 24 h after TBI. Nrf2 expression was detected by Western blot. Fucoxanthin treatment had no effect on improving motor performance (**C**), ameliorating brain edema (**D**) and reducing apoptotic cells (**E**) in Nrf2^−/−^ mice compared to the vehicle-treated group. n = 6 per group. ^@^*p* > 0.05 versus TBI + vehicle group. Scale bar: 50 mm.

**Figure 8:**
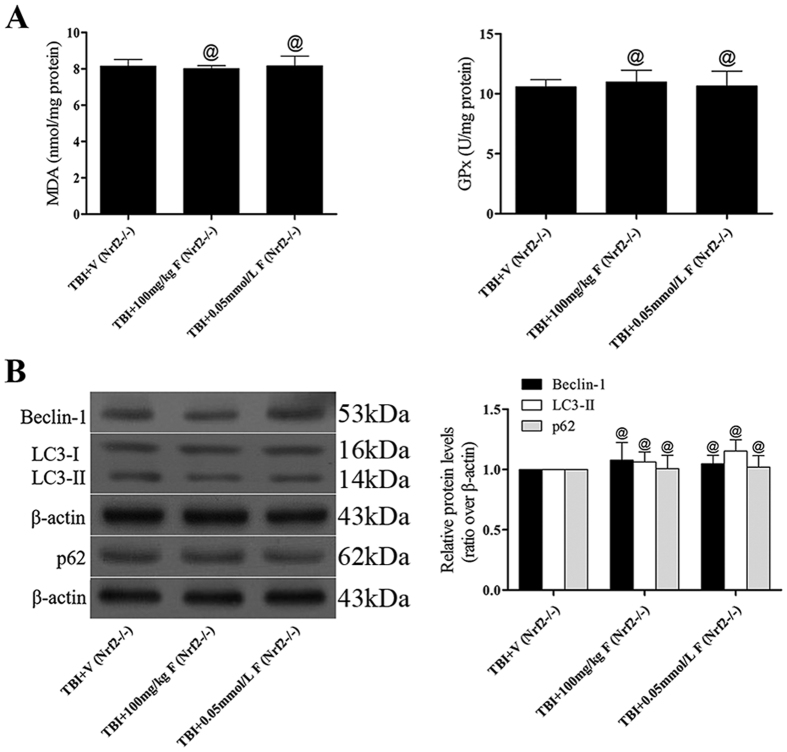
Fucoxanthin failed to suppress oxidative stress and activate autophagy in Nrf2^−/−^ mice following TBI. Fucoxanthin treatment had no effect on change the level of MDA and the activity of GPx (**A**) and the expression of Beclin-1, LC3 and p62 (**B**) in Nrf2^−/−^ mice compared to the vehicle-treated group. n = 6 per group. ^@^*p* > 0.05 versus TBI + vehicle group. β-actin was used as a loading control.

To prove our hypothesis that the neuroprotective and autophagy activation effects of fucoxanthin were attenuated in the absence of Nrf2, we evaluated the motor function, brain water content, neuron apoptotic index, antioxidant enzyme activity and the expression of Beclin-1, LC3, p62 in Nrf2^−/−^ mice subjected to TBI. As shown in Fig. 7A, nrf2-knockout mice-derived PCR products showed only one band of 400 bp, while wild-type mice-derived PCR products showed a band of 700 bp; both bands were seen in heterozygous mice-derived PCR products. Western blot analysis also validated the knockout efficiency, the expression of Nrf2 was obviously down-regulated in the Nrf2^(−/−)^ mice compared with the wild-type mice following TBI (Fig. 7B). Besides, our results showed that there were no significant differences of the grip test (*p* > 0.05, Fig. 7C) and brain water content (*p* > 0.05, Fig. 7D) between the vehicle-treated group and the fucoxanthin-treated group. Similarly, there were no significant differences in apoptotic index (*p* > 0.05, Fig. 7E), antioxidant enzyme activity (*p* > 0.05, Fig. 8A) and the expression of Beclin-1, LC3-II and p62 (*p* > 0.05, Fig. 8B), suggesting that fucoxanthin lost its neuroprotection in Nrf2^−/−^ mice and that the Nrf2-ARE and Nrf2-autophagy pathways were mechanisms involved in the neuroprotective role of fucoxanthin.

## Discussion

In the present study, we examined the neuroprotection of fucoxanthin and the possible role of Nrf2-ARE and Nrf2-autophagy pathways in the neuroprotective effects of fucoxanthin, in *in vivo* and *in vitro* models of TBI. The major findings were as follows: (1) Fucoxanthin provided neuroprotection after TBI; specifically, it improved neurobehavioral performance, alleviated brain edema and decreased lesion volume. (2) Fucoxanthin treatment decreased TBI-induced apoptosis and oxidative stress. (3) Fucoxanthin attenuated apoptosis and oxidative stress through activation of the Nrf2-ARE and Nrf2-autophagy pathways. These findings provide substantial evidence that fucoxanthin can provide neuroprotection against TBI, possibly, through activation of the Nrf2-ARE and Nrf2-autophagy pathways.

The secondary injury following TBI represents consecutive pathological processes including oxidative stress and apoptosis^17^. In the process of TBI, primary mechanical injury generates oxidants and their derivatives such as superoxide anions. These oxidants enhance the generation of ROS and exhausted antioxidant defense enzymes, such as catalase, superoxide dismutase and GPx, cause oxidative stress and lead to damage in lipid. The oxidative degradation of lipid could be reflexed by lipid peroxidation, and MDA formation is usually used as the index of lipid peroxidation^2^. In addition, these oxidants also increase mitochondrial depolarization and Ca^2+^ entry, then induce mitochondrial release of cytochrome c into the cytosol and sequential activation of caspase-3, caspase-9 and PARP, leading to apoptosis^18^. In our study, we found that apoptosis, MDA and GPx inactivation were induced after TBI but reversed by fucoxanthin. Possible mechanisms may be that TBI-induced oxidative stress contributes to the opening of mitochondrial membrane pores, which then leads to mitochondrial dysfunction and release of apoptosis-inducing factors such as cytochrome c and caspase-3. While fucoxanthin reduced TBI-induced apoptosis by regulation of oxidative stress.

In addition to the suppression of oxidative stress and apoptosis, we proposed another role of fucoxanthin, the induction of autophagy, which was confirmed by the formation of LC3 puncta, up-regulation of Beclin-1 and LC3-II as well as down-regulation of p62. Having documented that fucoxanthin activated autophagy in TBI, we further explored the role of autophagy in TBI-induced oxidative stress and apoptosis. Our data showed that inhibition of autophagy could attenuate fucoxanthin-induced suppression of oxidative stress and apoptosis, suggesting a protective role of autophagy. Consist with these observations showing that autophagy was benefit for TBI^19^,^20^, our results indicated that autophagy may serve as an additional target for adjuvant anti-oxidative stress and anti-apoptosis therapy.

The protective effects of fucoxanthin may involve multiple mechanisms. It has been suggested that fucoxanthin exhibited great therapeutic efficacy in Alzheimer’s disease by inhibiting acetylcholinesterase (AChE) and increasing brain-derived neurotrophic factor (BDNF) expression^21^. Besides, fucoxanthin could protect against visible light-induced retinal damage both *in vitro* and *in vivo* by reducing the generation of ROS^22^. While more recently, one study showed that in human keratinocytes (HaCaT), treatment of fucoxanthin augmented cellular antioxidant defense through activating Nrf2-driven expression of enzymes involved in glutathione synthesis and eliminated oxidative damage^23^. Nrf2 is a redoxsensitive, basic leucine zipper protein that regulates the transcription of several antioxidant genes. It has been widely studied in TBI models during the past decades. Evidence have demonstrated that Nrf2 and its downstream ARE-containing genes such as HO-1 and NQO-1 were activated after TBI^24^. Moreover, Nrf2^−/−^ mice exhibited poorer outcomes than wild-type mice, and administration of histone deacetylase inhibitors or tBHQ may protect against TBI through activation of Nrf2^25^. These studies proposed that activation of the Nrf2-ARE pathway is beneficial for TBI. Our observations were consistent with these reports, showing that fucoxanthin could regulate this antioxidant pathway by promoting Nrf2 translocation from cytoplasm to nucleus.

To confirm that the neuroprotection, the inhibition of apoptosis and oxidative stress, and the induction of autophagy by fucoxanthin was through Nrf2, we employed the Nrf2 gene knockout mice. The data showed that the neuroprotective effects were significantly decreased without Nrf2. Moreover, the Nrf2 knockout reversed fucoxanthin-induced the inhibition of apoptosis and oxidative stress as well as the activation of autophagy after TBI. Several lines of evidence have also indicated that the defective activities of antioxidant enzymes and exacerbated injury were existed without Nrf2^26^,^27^. A probable mechanism by which fucoxanthin regulates Nrf2/ARE pathway has not been fully explained. Liu *et al*. found that in murine hepatic BNL CL.2 cells, fucoxanthin significantly increased intracellular ROS generation, ROS produced by fucoxanthin activated the mitogen-activated protein kinase (MAPK) pathway to induce Nrf2 phosphorylation, resulting in the dissociation of Nrf2 from its repressor keap1 and its translocation into nucleus^28^. Therefore, it can be concluded that the indirect activation of Nrf2-ARE signaling by fucoxanthin may, at least in part, be due to its pro-oxidant activity, which then activated upstream protein kinases of Nrf2, such as MAPK pathway. The pro-oxidant activity of fucoxanthin may arise from the 5,6-monoepoxide, a unique structure of fucoxanthin, which has been shown to undergo ring-opening reactions as a result of attacking nucleophiles^29^. However, in the study conducted by Liu *et al*., fucoxanthin promoted the generation of ROS, while in our study, fucoxanthin suppressed ROS production, indicating that additional signaling pathways may be involved. It has been proposed that fucoxanthin may directly cause Nrf2 translocation by reacting with the thiol groups of Keap1 through Michael addition because of the presence of an α, β-unsaturated carbonyl group in its structure^28^. Fucoxanthin has also been reported to indirectly activated Nrf2/ARE pathway via AKT pathways. AKT is a classic signal-transducing protein which can activate the primary cellular defense mechanism Nrf2/ARE in cells^30^. Zheng *et al*. found that in human keratinocytes, fucoxanthin increased the level of phosphorylated AKT and elevated the nuclear level of Nrf2. Furthermore, LY294002, a specific inhibitor of AKT, significantly suppressed the active form of AKT, which resulted in reduction of Nrf2 accumulation^23^. Thus, in TBI models, fucoxanthin may activate the Nrf2/ARE pathway by directly reacting with the thiol groups of Keap1 or indirectly activating AKT pathway. While in other models, fucoxanthin may also activate the Nrf2/ARE pathway by indirectly promoting ROS generation. However, these are just our hypothesis and further researches are needed to clarify them.

How Nrf2 regulates autophagy has still not been entirely clear. There have been several explanations, and these explanations were consistently related to p62. p62 is a substrate for lysosomal proteases, it is a protein possessing dual-binding sites for ubiquitin chains and LC3. p62 binds to LC3 via an LC3-interacting region (LIR) and ubiquitin chains through a UBA (ubiquitin-associated) domain, subsequently leading to the activation of autophagy^31^. Therefore, stimuli such as hypoxia and amino acid deprivation have been shown to induce autophagy as well as p62 degradation and, subsequently, decrease p62 intracellular levels^32^. Puissant and his colleagues suggested that Nrf2 could specifically binds to the antioxidant-responsive element located in the p62 promoter to promote the expression of p62, which then combines with LC3 and activates autophagy^33^. Moreover, another research showed that Keap1 uncoupled from Nrf2 can bind to p62, interact with LC3 and transport the ubiquitin conjugate to the autophagosome for degradation^34^. While how Nrf2 regulated autophagy in TBI models is unclear, further studies are needed to clarify it.

In conclusion, our study indicated that fucoxanthin exerted neuroprotection against TBI by combating apoptosis and oxidative stress, at least partly via Nrf2-ARE and Nrf2-autophagy pathways. These results make fucoxanthin an attractive therapeutic agent in treatment of TBI in the future.

## Materials and Methods

### Animals

The experiment protocols in this study including animal usages and the surgical procedures are abided by the Guide for the Care and Use of Laboratory Animals by the National Institutes of Health (NIH) and approved by the Animal Care and Use Committee of Nanjing University. Male ICR mice (Experiment Animal Centre of Jinling hospital, Jiangsu, China) and Nrf2-deficient (Nrf2^−/−^) mice (with permission from Dr. Thomas W. Kensler, Johns Hopkins University, Baltimore, MD, USA) weighing 28–32 g were used in this study. Mice were housed on a 12 h light/dark cycle at 23 ± 1 °C with free access to food and water.

### Primary culture of mouse cortical neurons

The primary culture of mouse cortical neurons was prepared as described previously^35^. Briefly, cortical neurons were isolated from the embryos of time-mated pregnant mice and then cultured on poly-D-lysine-coated 6-well dishes at a density of 1 × 10^6^ cells per well. The primary mouse cortical neurons were cultured in neurobasal medium (Life Technologies, Carlsbad, CA, USA; catalog number: 21103049) supplemented with 1 mM glutamate (Sigma-Aldrich, St.Louis, Mo, USA; catalog number: G3291) and 2% B27 (Life Technologies, Carlsbad, CA, USA; catalog number: 17504044) at 37 °C and 5% CO_2_ incubator. Every 3 days, half of the culture medium was replaced with fresh medium. The *in vitro* studies were performed after culture of 10–12 days.

### Models of TBI

The *in vivo* model of TBI employed in the present study was based on a weight-drop model described previously^36^. In brief, mice were anesthetized with chloral hydrate (1%, 5 ml/kg) intraperitoneal (i.p.) injection and placed onto the platform under the weight of the weight-drop device. Then we made a 1.5 cm midline longitudinal scalp incision and exposed the skull. The left anterior frontal area (1.5 mm lateral to the midline on the mid-coronal plane) was located as the impact area and a 200-g weight was released onto the skull from a height of 2.5 cm. The mortality rate by apnea was decreased via early respiratory support and the scalp wound was sutured by using standard suture material. Injured mice were returned to cages with free access to food and water. The sham-injured mice underwent the same procedures, however did not undergo the weight drop.

The *in vitro* model of TBI was performed according to a previous study^35^. In brief, every 6-well plate was scratched with a sterile plastic needle followed by a 9 × 9 square grid (the space between the lines was 4 mm) manually. Neuronal cells were then cultured for another 24 h at 37 °C and 5% CO_2_ incubator without change of medium. Un-scratched cells were used as control.

### Groups and drug administration

For *in vivo* studies, mice were divided into 9 groups randomly: sham, TBI, TBI + vehicle, TBI + fucoxanthin intragastric (i.g.) administration (three different dose groups: 50 mg/kg, 100 mg/kg, 200 mg/kg) and TBI + fucoxanthin intracerebroventricular (i.c.v.) injection (three different dose groups: 0.01 mmol/L, 0.05 mmol/L, 0.1 mmol/L).

Fucoxanthin was administered into mice by two methods. Firstly, fucoxanthin (3 different doses) was given by i.g. administration. Fucoxanthin (Sigma-Aldrich, St.Louis, Mo, USA; catalog number: F6932) was diluted in olive oil (1 ml/kg) immediately before use. The doses used in this section was based on a study of fucoxanthin in an acetylcholinesterase model^21^. Secondly, fucoxanthin was administered by i.c.v. injection in 3 different doses 30 minutes after TBI. The i.c.v. injection was selected because our study is the first report to explore the role of fucoxanthin in brain injury models and there have no reports showing that fucoxanthin can cross blood brain barrier (BBB), thus we administrated fucoxanthin by i.c.v. injection. The doses used in this section (0.01 mmol/L, 0.05 mmol/L and 0.1 mmol/L) was according to our preliminary experiment. Fucoxanthin was prepared in saline with 10% dimethyl sulphoxide (DMSO). Fucoxanthin or vehicle was i.c.v. injection (right lateral ventricle; 0.1 mm posterior and 1.0 mm lateral of the bregma, 3.0 mm in depth) with a Hamilton syringe 30 min after TBI. An equal amount of 10% DMSO was administered in the sham and TBI + vehicle groups. 3-Methyladenine (3-MA) (Sigma-Aldrich, St. Louis, Mo, USA; catalog number: M9281) was dissolved in saline with 10% DMSO. 3-MA was injected i.c.v. 30 min before TBI. The dose of 3-MA (400 nM) were based on prior investigations in the model of mouse TBI and our preliminary experiments.

For *in vitro* experiments, neuronal cells were divided into following groups: control, TBI, TBI + DMSO, TBI + edaravone and TBI + fucoxanthin (three different dose groups: 5 μM, 10 μM and 20 μM). Edaravone and fucoxanthin were first dissolved in DMSO and then added to cultured media to reach different final concentrations. The concentrations of edaravone and fucoxanthin used in *in vitro* study were according to previous studies^23^,^37^ and our preliminary experiments.

### Neurological deficit and brain water content

The neurologic status of the mice was evaluated at 1 day, 3days and 7 days after TBI using the NSS and grip test^38^. For NSS, the investigators evaluate the ability of each mouse to perform 10 different tasks that demonstrate motor function, balance, and alertness. One point is given for failing to perform each of the tasks; thus 0 = minimum deficit and 10 = maximum deficit. The scoring system was adapted from previously published methods^39^. A detailed description of our NSS testing procedure and the contribution of individual tasks to the composite NSS was published previously^36^. For grip test, mice were placed on a thin, horizontal, metal 45 cm long wire which was lay up between two vertical poles 45 cm above a foam pad. Zero point was given if the mice was unable to remain on the wire for less than 30 s; one point was given if the mice failed to hold on to the wire with both hind paws and forepaws together; two points were given if the mice held on to the wire with both hind paws and forepaws but not the tail; three points were given if the mice used its tail along with both hind paws and forepaws; four points were given if the mice moved along the wire on all four paws plus tail; five points were given if mice that scored four points also ambulated down one of the posts used to support the wire. The grip test was performed in tree times and a total point was calculated for each mouse. The test was carried out by an investigator who was blinded to the experimental groups.

The brain water content was employed as described previously^40^. Briefly, mouse brain was taken out and placed onto a cooled brain matrix 1 day after TBI. The brain cerebellum and stem were taken away and the ipsilateral tissue was weighed immediately to obtain the wet weight (ww). Subsequently, the hemisphere was dried for 72 h at 80 °C and weighed to obtain the dry weight (dw). The brain water content equals (ww−dw)/ww × 100%.

### Tissue processing

Mice were deeply anesthetized with chloral hydrate 24 h after TBI and perfused intracardially with 30–40 ml of cold (4 °C) heparinized 0.9% saline. For western blot, biochemical measurements, electrophoresis mobility shift assay (EMSA) and realtime quantitative polymerase chain reaction (RT-PCR), the left (ipsilateral) cerebral cortex peri-contusion was collected and frozen in liquid nitrogen immediately and then stored in a −80 °C freezer until use. For immunohistochemistry, immunofluorescence and Terminal deoxynucleotidyl transferase-mediated dUTP nick 3′-end labeling (TUNEL) staining, the whole brain was collected and soaked in 4% paraformaldehyde overnight.

### Lesion volume measurements

The measurement of lesion volume was according to previous study^41^. Briefly, 7 days after TBI, all animals were anesthetized using a solution of chloral hydrate and sacrificed. The brains were removed, post-fixed for 12 h, and then cryoprotected in 15% sucrose in PBS. Tissue damage was determined by morphometric image analysis. Brain sections obtained at 500-mm intervals spanning the length of the brain were stained with cresyl violet. The areas of the lesion, injured and non-injured hemisphere, and cortex were determined using an image analysis system. Area measurements from each tissue section were obtained and summed, and corresponding volumes were calculated. Lesion volume was quantitatively analyzed with Image-Pro Plus 6.0 and was expressed as % (the percentage volume of the non-injured hemisphere) as described previously.

### Determination of malondialdehyde (MDA) and glutathione peroxidase (GPx) activity

The cerebral cortex tissue was homogenized in 2 ml of 10 mM phosphate-buffer (pH 7.4). Following centrifugation for 20 min at 12,000 g, the content of MDA and the activity of GPx in the supernatant was evaluated by a spectrophotometer (Nanjing Jiancheng Biochemistry Co., Nanjing, China). The Bradford method was used to determine the protein concentrations.

### Immunohistochemical staining

For immunohistochemical staining, the tissue sections (4 mm) were incubated with Nrf2 antibody (1:100, Abcam, Cambridge, MA, USA; catalog number: ab31163) overnight at 4 °C. After washing with phosphate buffer solution (PBS) for three times (5 min per wash), the sections were incubated with horseradish peroxidase-conjugated IgG (1:400, Santa Cruz Biotechnology, Santa Cruz, CA; catalog number: sc-2748) for 60 min. Diaminobenzidine (DAB) was used to visualize immunolabellin with haematoxylin counterstaining.

### Western blot analysis

Western blot was performed as described previously^36^, equal amounts of protein were separated by 10% sodium dodecyl sulfate-polyacrylamide gel electrophoresis (SDS-PAGE), transferred to polyvinylidene fluoride (PVDF) membranes and then incubated with corresponding primary antibodies overnight at 4 °C. The antibodies used were caspase-3 (1:1000; Cell Signaling Technology, Danvers, MA, USA; catalog number: 9661), poly (ADP-ribose) polymerase (PARP) (1:1000; Cell Signaling Technology, Danvers, MA, USA; catalog number: 9542), COX IV (1:1000; Cell Signaling Technology, Danvers, MA, USA; catalog number: 11967), histone H3 (1:1000; Cell Signaling Technology, Danvers, MA, USA; catalog number: 9715), p62/SQSTM1 (1:1000; Abcam, Cambridge, MA, USA; catalog number: ab109012), cytochrome c (1:5000; Abcam, Cambridge, MA, USA; catalog number: ab133504), Nrf2 (1:1000; Abcam, Cambridge, MA, USA; catalog number: ab31163), HO-1 (1:2000; Abcam, Cambridge, MA, USA; catalog number: ab13243), NQO-1 (1:1000; Abcam, Cambridge, MA, USA; catalog number: ab34173), Beclin-1 (1:1000; Novus Biological, Littleton, CO, USA; catalog number: NB500-249), microtubule associated protein 1 light chain 3 (LC3) (1:1000; Novus Biological, Littleton, CO, USA; catalog number: NB600-1384) and β-actin (1:5000; Bioworld Technology, Minneapolis, MN, USA; catalog number: AP0060). Subsequently, the membranes were incubated with corresponding secondary antibodies for 2 h at room temperature.

### Real-time quantitative polymerase chain reaction (RT-PCR)

Total RNA was extracted from tissue samples by RNAiso Plus (TaKaRa Bio., Dalian, China; catalog number: 9108) and reverse-transcribed to cDNA by PrimeScript RT reagent kit immediately (TaKaRa Bio. catalog number: RR037A). The primer sequences were as follows: HO-1: F, 5′-ATCGTGCTCGCATGAACACT-3′; R, 5′-CCAACACTGCATTTACATGGC-3′; NQO-1: F, 5′-CATTCTGAAAGGCTGGTTTGA-3′; R, 5′-CTAGCTTTGATCTGGTTGTCAG-3′; β-actin: F, 5′-AGTGTGACGTTGACATCCGTA-3′; R, 5′- GCCAGAGCAGTAATCTCCTTCT-3′. The primers were designed based on PubMed GenBank and synthesized by Invitrogen Life Technologies (Shanghai, China). RT-PCR analysis was performed using the Mx3000 P System (Stratagene, San Diego, CA, USA). β-actin was used as an endogenous reference “housekeeping” gene.

### Immunofluorescence staining

The level of autophagy was assessed according to a previous immunostaining protocol as follows: the slides of each coronal section were incubated in blocking buffer for 2 h, and then washed three times with PBS for 10 min. Afterward, incubated with anti-LC3 antibody (1:200; Novus Biological, Littleton, CO, USA; catalog number: NB600-1384), the slides kept overnight in a dark place at 4 °C. Subsequently, washed three times with PBS, the slides were incubated with anti-NeuN antibody (1:100; Cell Signaling Technology, Danvers, MA, USA; catalog number: 24307) under similar conditions. The following day, thoroughly washed three times with PBS, the slides were incubated with the corresponding secondary antibodies for 1 h at room temperature. After washing three times with PBS, the slides were stained with 2-(4-amidinophenyl)-6-indolecarbamidine dihydrochloride (DAPI; Beyotime Biotech Inc., Nantong, China; catalog number: C1002) for 2 min to show the locations of nuclei. Then, coverslips were used with the fluorescence quenching agent. We imaged the fluorescently stained cells via Olympus IX71 inverted microscope system and analyzed using Image-Pro Plus 6.0 software (Media Cybernetics, Silver Spring, MD).

### TUNEL staining

TUNEL staining was performed using an *In Situ* Cell Detection Kit (Roche, South San Francisco, CA, USA; catalog number: 11684817910) according to the manufacturer’s instructions. Briefly, the sections were incubated with labeling solution containing TUNEL for 60 min at 37 °C. Deoxyribonucleic acid (DNA) was visualized with a 1:40 dilution of streptavidin peroxidase (HRP) and staining with DAB as chromogen. For quantification, six random vision fields (400x) surrounding contusion in each section were chosen, the mean number of TUNEL-positive cells in the six views was regarded as the data of each section and a total of four sections from each animal were used for quantification. The final average number of the four sections was regarded as the data for each sample. Apoptotic index, called as the average percentage of apoptotic cells, was detected to evaluate the extent of brain damage. All the processes were conducted by two independent observers who had no prior known the group assignments.

### Nuclear extraction and electrophoresis mobility shift assay (EMSA)

Nuclear extraction was prepared following the manufacturer’s instructions of the Nuclear and Cytoplasmic Protein Extraction Kit (Beyotime Biotech Inc., Nantong, China; catalog number: P0028). EMSA was performed with a commercial kit (Beyotime Biotech Inc., Nantong, China; catalog number: GS008) to assay Nrf2-DNA binding activity^42^. Consensus oligonucleotide probes for Nrf2 (5′-TGGGGAACCTGTGCTGAGTCACTGGAG-3′) were end-labeled with biotin. Reactions were performed in the presence of 10 μg of nuclear extract and incubation buffer (10 mM Tris, pH 7.5, 5 mM MgCl_2_, 50 mM KCl, 0.05% Nonidet P-40, 2.5% glycerol, 1 mM dithiothreitol and 1 μg poly(dI–dC)) at room temperature for 20 min. The mixture was loaded on a 6% precast polyacrylamide gel in 0.5 × Tris-borate-EDTA (TBE) at 100 V for 1 h and transferred to a nylon membrane in 0.5 × TBE at 100 V for 30 min. Transferred DNAs were cross-linked to the membrane and detected using horseradish peroxidase-conjugated streptavidin.

### Cell viability analysis

Primary neuronal cell viability was assessed by the lactate dehydrogenase (LDH) activity according to the manufacturer’s instructions (Beyotime Biotech Inc., Nantong, China; catalog number: C0016)^43^. Neuronal cells were treated with LDH release agent and the media containing detached cells were collected and centrifuged. The supernatant was used to examine LDH activity. The OD value at 490 nm was measured by a spectrophotometer. The percentage of damaged cells = (OD490_sample_ − OD490_media_)/(OD490_maximum_ − OD490_media_) × 100%. OD490_media_ = media without cells and OD490_maximum_ = cells treated with LDH release agent.

### Measurement of intracellular reactive oxygen species (ROS)

The intracellular ROS levels were analyzed using 2′, 7′-dichlorodihydrofluorescein diacetate (DCFH-DA, Beyotime Biotech Inc., Nantong, China; catalog number: S0033). Briefly, cells were washed with PBS, centrifuged at 1000 rpm for 5 min twice and incubated with 10 μM DCFH-DA for 10 min. After rinsing with PBS, cells were transferred to a 96-well black plate. Fluorescence was measured by a fluorescence spectrophotometer (Thermo Scientific Varioskan Flash, USA) at an excitation wavelength of 488 nm and an emission wavelength of 525 nm.

### Identification of Nrf2 genotypes

Breeding pairs of Nrf2-deficient ICR mice were kindly provided by Dr. Thomas W. Kensler (Johns Hopkins University). Genotypes of Nrf2^−/−^ and Nrf2^+/+^ mice were confirmed by PCR amplification of genomic DNA isolated from the tail tissue. PCR amplification was carried out using three different primers: 5′-CGCCTTTTCAGTAGATGGAGG-3′(antisense for wild-type); 5′-GCGGATTGACCGTAATGGGATAGG-3′ (antisense for LacZ); 5′-TGGACGGGACTATTGAAGGCTG-3′ (sense for both genotypes),

### Statistical analysis

SPSS 19.0 software package (SPSS Inc., Chicago, IL, USA) was used for the statistical analysis. Statistical analysis between two groups were performed with the Student’s t test and between multiple groups with one-way ANOVA followed by Tukey’s test. Every experiment was repeated at least three times and the data are reported as the mean ± SEM. A value of p < 0.05 was considered statistically significant.

## Additional Information

**How to cite this article**: Zhang, L. *et al*. Fucoxanthin provides neuroprotection in models of traumatic brain injury via the Nrf2-ARE and Nrf2-autophagy pathways. *Sci. Rep.* **7**, 46763; doi: 10.1038/srep46763 (2017).

**Publisher's note:** Springer Nature remains neutral with regard to jurisdictional claims in published maps and institutional affiliations.
